# Impact of sodium alginate‐based film loaded with resveratrol and thymol on the shelf life of cooked sausage and the inoculated *Listeria monocytogenes*

**DOI:** 10.1002/fsn3.3702

**Published:** 2023-09-23

**Authors:** Mahsa Hashemi, Majid Aminzare, Hassan Hassanzadazar, Shahin Roohinejad, Reza Tahergorabi, Alaa El‐Din Ahmed Bekhit

**Affiliations:** ^1^ Student Research Committee, Department of Food Safety and Hygiene, School of Public Health Zanjan University of Medical Sciences Zanjan Iran; ^2^ Department of Food Safety and Hygiene, School of Public Health Zanjan University of Medical Sciences Zanjan Iran; ^3^ Division of Food and Nutrition, Burn and Wound Healing Research Center Shiraz University of Medical Sciences Shiraz Iran; ^4^ Food and Nutritional Sciences Program North Carolina Agricultural and Technical State University Greensboro North Carolina USA; ^5^ Department of Food Science University of Otago Dunedin New Zealand

**Keywords:** bioactive compound, chemical stability, edible packaging, herbal preservative, microbial safety

## Abstract

In present study, sodium alginate biodegradable films containing different concentrations of resveratrol (RES: 0.002% and 0.004%) or thymol (THY: 0.5% and 1%) and their combinations were prepared, and evaluated for their effects on spoilage‐related microbial profile, lipid oxidation, sensory properties, and protective effects against *Listeria monocytogenes* in beef mortadella sausage during 40 days storage at 4°C. The release rate of phenolic compounds was determined by the Folin–Ciocalteu test. To assess the shelf life of the product, changes in total viable count (TVC), lactic acid bacteria count (LAB), psychrotrophic bacteria count (PTC), pH levels, thiobarbituric acid reactive substances (TBARS) levels, and sensory characteristics (taste, color, odor, and overall acceptability) were evaluated. For the sensory evaluation, a panel of 70 semi‐trained judges was selected according to their initial performance. Samples wrapped with sodium alginate films containing 1% THY (alone or combined with different concentrations of RES) exhibited lower bacterial counts compared to other experimental groups at the end of the storage period (6.01–6.35 vs. 6.71–8.17 log_10_ CFU/g for TVC, 5.37–5.83 vs. 6.07–7.11 log_10_ CFU/g for LAB, 5.08–5.18 vs. 5.40–7.23 log_10_ CFU/g for PTC, and 6.53–6.92 vs. 7.23–9.01 log_10_ CFU/g for inoculated *L. monocytogenes*). Sodium alginate films containing the combination of 0.004% RES and different concentrations of THY showed higher antioxidant effects than other experimental groups (TBARS values of 1.68–1.99 vs. 2.23–3.80 mg MDA/kg sample). The sodium alginate film containing 0.004% RES + 1% THY exhibited the highest antimicrobial and antioxidant activities and highest sensory scores among all treatments. These findings highlight the potential application of the sodium alginate film containing a combination of RES and THY as an active packaging material with natural preservatives in the meat products industry. This application can effectively extend the shelf life and enhance the microbial safety of clean‐label cooked sausages during refrigerated storage.

## Graphical abstract

Alginate films containing different concentrations of RES and/or THY were prepared. The effects of sodium alginate films on the shelf life and microbial safety of sliced beef mortadella sausage during 40 days of storage at 4°C were assessed. Sliced beef mortadella sausage wrapped with 0.004% RES + 1% THY film showed the best microbial, chemical, and sensory properties.

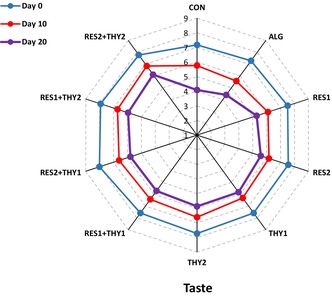

## 1 INTRODUCTION

Meat and meat products are recognized as a significant component of the modern diet owing to their high protein content with a high biological value and various essential micronutrients (Pateiro et al., 2021; Umaraw et al., 2020). Cooked sausages (such as beef mortadella sausage) are highly prone to lipid oxidation and microbial spoilage due to the use of ground meat and its high lipid and protein contents (Umaraw et al., 2020). In this regard, significant destructive changes can occur during processing steps, especially after heat treatment, such as cutting and packaging (Tajbakhsh et al., 2023). In addition, improper handling and poor post‐processing storage can contaminate these types of meat products with important foodborne pathogens, such as *Listeria monocytogenes*. Due to the increased mortality rate associated with listeriosis, introducing biological control agents is necessary to ensure food safety against *L. monocytogenes* (Pateiro et al., 2021; Zamuz et al., 2021).

Utilizing antimicrobial and antioxidant compounds is a promising strategy to extend the shelf life and improve the microbial safety of meat products. The growing awareness of health risks associated with synthetic additives has led to a rising preference among consumers for natural alternatives (Raji et al., 2019; Zarei et al., 2022). In this regard, some studies previously evaluated the antimicrobial and antioxidant effects of plant‐based preservatives on cooked sausage (Alirezalu et al., 2020; Pateiro et al., 2021) including beef mortadella sausage (Biasi et al., 2023; Martins et al., 2019). Thymol (THY: 2‐isopropyl‐5‐methylphenol) is a natural monoterpenoid phenol found as the main bioactive compound in essential oils extracted from thyme, oregano, and some other medicinal plants. This volatile compound is known for its antioxidant and antimicrobial properties, and it has been designated as a generally recognized safe food additive by the Food and Drug Administration (FDA). However, the use of THY may cause allergic reactions such as dermatitis or skin inflammation in some people. Regarding thyme and THY toxicity, despite their generally recognized as safe (GRAS) status, it is recommended to limit thyme consumption to 10 g of dried leaves (0.03% phenols calculated as THY) per day to prevent potential toxicity. Studies underscore the potential implications of THY's presence and its metabolites in improving human health. These studies have shown that free THY could not be detected in human plasma or urine after oral intake. Alternatively, THY was present in human plasma as THY sulfate and as both THY sulfate and THY glucuronide in urine (Salehi, Mishra, Shukla, et al., 2018). Resveratrol (RES: trans‐3,4′,5‐trihydroxystilbene) is a polyphenol obtained from diverse groups of plants, especially in red wine, muscadine grape, cranberry, lingonberry, and redcurrant. The antioxidant and antimicrobial properties of RES have been reported in several studies (Sharifi‐Rad et al., 2022). RES is generally safe in short‐term doses of 1 g per day, but some studies have shown that doses of 2.5 g or more per day can lead to side effects such as nausea, vomiting, diarrhea, and liver dysfunction in patients with non‐alcoholic fatty liver disease. However, some other studies have found that up to 5 g of RES per day is safe and tolerable (Salehi, Mishra, Nigam, et al., 2018). The oral absorption of RES in humans is approximately 75% and is primarily believed to happen through transepithelial diffusion. Extensive metabolism in the intestines and liver leads to an oral bioavailability of significantly less than 1%. Metabolic studies, both in plasma and in urine, have revealed major metabolites to be glucuronides and sulfates of RES (Jayan et al., 2019). In order to obtain comparable effects to synthetic preservatives and prevent adverse sensory effects or even toxicity issues in meat products, the combined use of bioactive components at lower concentrations in active packaging systems is a suitable strategy (Pateiro et al., 2021). The utilization of synthetic polymers in active food packaging films can give rise to human and environmental issues (Mignon et al., 2019). In this context, biodegradable polymers, such as edible films, are a promising alternative to synthetic polymers (Paidari et al., 2021; Umaraw et al., 2020). Several studies have been conducted on the antimicrobial and antioxidant effects of active food packaging systems containing plant preservatives on different foods (Bagher Abiri et al., 2023; Chaari et al., 2022; Moosavi‐Nasab et al., 2023; Noorbakhesh & Danaee, 2021; Smaoui et al., 2022; Yadav et al., 2022). Some of these studies have specifically focused on incorporating THY or RES into biodegradable films or coatings (Abdalbeygi et al., 2022; Ansarian et al., 2022; Bazargani‐Gilani & Pajohi‐Alamoti, 2020; Hassan & Cutter, 2020; Karam et al., 2019). Sodium alginate, as the initial by‐product of algae purification, is an economical, non‐toxic, and biodegradable hydrocolloid. It possesses the ability to create flexible and durable films and coatings (Saei et al., 2021). According to previous studies, these films have the potential to replace conventional films in the meat industry as a basic component of packaging materials. They can extend the shelf life of meat and meat products by preventing dehydration, rancidity, or browning of the muscle tissue. In addition, the sodium alginate film can include various natural preservatives (such as RES and THY) as an active packaging material. Composite or multilayer films based on sodium alginate can be prepared by casting or extrusion methods for use in the meat packaging industry. These edible films also exhibit low permeability to both oxygen and oil, making them suitable for the meat and meat products packaging industry. The FDA has categorized alginates as GRAS substances (Gheorghita et al., 2020).

Several studies have assessed the impact of biodegradable films containing various plant‐based essential oils and/or extracts on the microbial quality, oxidative stability, sensory attributes, and/or microbial safety aspects of sliced cooked sausages (Khodayari et al., 2019; Moradi et al., 2011; Rezaeigolestani et al., 2017; Ruiz‐Navajas et al., 2015). However, to the best of our knowledge, no comprehensive research has investigated the antimicrobial, antioxidant, and sensory effects of biodegradable films containing pure RES and/or THY phenolic compounds in extending the shelf life and enhancing the microbial safety of cooked beef mortadella. Therefore, the aim of the present study was threefold: (i) to prepare sodium alginate films containing different concentrations of RES and/or THY and evaluate the release phenolic compounds rate to product, (ii) to assess the effects of sodium alginate films on various shelf‐life indices (including spoilage‐related microbial profile, lipid oxidation, and sensory characteristics) of sliced beef mortadella sausage during 40 days of storage at 4°C, and (iii) to investigate the impact of sodium alginate films on the microbial safety of sliced mortadella sausage through inoculation with *Listeria monocytogenes*.

## 2 MATERIALS AND METHODS

### 2.1 Materials

Sodium alginate powder, resveratrol powder (3,4′,5‐Trihydroxy‐*trans*‐stilbene), thymol powder, glycerol, Tween 80, Folin–Ciocalteu reagent, gallic acid, sodium carbonate (Na_2_CO_3_), butylated hydroxytoluene (BHT), calcium chloride (CaCl_2_), 1,1,3,3‐tetraethoxypropane (TEP), 2‐thiobarbituric acid (TBA), and ethanol were purchased from Sigma‐Aldrich Company. The filter paper was purchased from Whatman International Ltd.. Plate Count Agar (PCA), Peptone water (PW), de Man Ragosa Sharpe (MRS) agar, Rose Bengal Chloramphenicol (RBC) agar, Listeria Chrom agar, perchloric acid, and GasPak System (type C) were purchased from Merck Company. A lyophilized vial of a pathogenic strain of *L. monocytogenes* PTCC 1783 was purchased from the microbial collection of the Iranian Research Organization for Science and Technology (IROST). All chemicals and reagents used in this study were food grade or reagent grade.

### 2.2 Preparation of sodium alginate films containing RES and/or THY

Sodium alginate films were prepared as described by Pavli et al. (2019) with some modifications. Briefly, 2 g of sodium alginate powder and 0.01 g of CaCl_2_ were dissolved in 100 mL of sterilized distilled water. Then, 1.5% (v/v) glycerol was added to the film‐forming solutions (FFSs) as a plasticizer. Different concentrations of RES (0, 0.002, and 0.004% w/v) and THY (0, 0.5, and 1% w/v), either individually or combined, were added to the FFSs. The concentrations of RES and THY selected for this study were determined through preliminary in vitro trials, which included assessing the minimum inhibitory concentration (MIC) as well as evaluating the 2,2‐diphenyl‐1‐picrylhydrazyl (DPPH) radical scavenging activity across various random concentrations of RES and THY (data not shown). Tween 80 was also used as an emulsifier in the same concentrations as RES and THY. FFSs were dried into Teflon plates and resulting films were exposed to ultraviolet light, and stored in sterile low‐density polyethylene bags until testing. A list of sodium alginate films containing different concentrations of RES and THY used in the present study is shown in Table 1.

**TABLE 1:** List of sodium alginate films containing different concentrations of resveratrol and/or thymol.

No.	Treatment	Description
1	ALG	Sodium alginate film
2	RES_1_	Sodium alginate film containing 0.002% (w/v) resveratrol
3	RES_2_	Sodium alginate film containing 0.004% (w/v) resveratrol
4	THY_1_	Sodium alginate film containing 0.5% (w/v) thymol
5	THY_2_	Sodium alginate film containing 1% (w/v) thymol
6	RES_1_ + THY_1_	Sodium alginate film containing 0.002% (w/v) resveratrol + 0.5% (w/v) thymol
7	RES_2_ + THY_1_	Sodium alginate film containing 0.004% (w/v) resveratrol + 0.5% (w/v) thymol
8	RES_1_ + THY_2_	Sodium alginate film containing 0.002% (w/v) resveratrol + 1% (w/v) thymol
9	RES_2_ + THY_2_	Sodium alginate film containing 0.004% (w/v) resveratrol + 1% (w/v) thymol

### 2.3 Application of sodium alginate films on mortadella sausage

The overall goal of the research is to use the new active packaging material under meat‐slicing conditions. Therefore, beef mortadella sausage slices (12.5 g weight and 2 mm thickness) were purchased from a local supplier and transported to the laboratory under aseptic conditions in polystyrene boxes containing ice packs. The ingredients used in the sausage based on the traditional formula included beef, vegetable oil, water, potato starch, flour, garlic, sodium chloride, mixed spices, sodium ascorbate, sodium tripolyphosphate, and sodium nitrate. The proximate composition of beef mortadella samples, including moisture, lipids, protein, total carbohydrates (based on starch), and ash, was 53.3%, 22.7%, 12.4%, 8.6%, and 3.0%, respectively. The upper and lower surfaces of the sausage slices were wrapped with different films and transferred separately to sterile low‐density polyethylene bags under aseptic conditions. Sausage samples without any sodium alginate films were used as the control group (CON). All experimental groups were stored at 4°C before sampling for analysis at 0, 10, 20, 30, and 40 days (Moradi et al., 2011). The experiment was repeated three times using independent samples.

### 2.4 Release of phenolic compounds from sodium alginate films into mortadella sausage

On each sampling day, films were removed from the middle of two sausage slices, and the total phenolic content of the films was determined using the Folin–Ciocalteu method (Moradi et al., 2011). For this purpose, 25 mg of each sodium alginate film was blended in 3 mL of distilled water for 5 min. Then, 0.1 mL of the film extract was combined with 7 mL of distilled water and 0.5 mL of Folin‐Ciocalteu reagent. The resulting mixture was kept at 25°C for 8 min. Then, distilled water and 1.5 mL of Na_2_CO_3_ (2% w/v) were added to the mixture, bringing the final volume to 10 mL. The mixture was then incubated in the dark at 25°C for 2 h and the absorbance of the mixture was measured at 765 nm by a spectrophotometer (DR 5000; HACH Co.). The calibration curve was generated using different concentrations of gallic acid and the results were computed using the formula provided below:

$$T=\frac{\mathrm{CV}}{M}$$

where *T* represents the total phenolic content (mg GAE/g film), *C* denotes the concentration of gallic acid derived from the calibration curve (mg/mL), *V* represents the volume of the film extract (mL), and *M* represents the weight of the dried film (g).

### 2.5 Effects of sodium alginate films on the shelf life of mortadella sausage

#### 2.5.1 Evaluation of microbial spoilage

Sausage samples weighing 12.5 g were aseptically put in sterile stomacher bags with 112.5 mL of PW (0.1% v/v) and homogenized using a stomacher blender (Seward Stomacher 400 Circulator), with 400 strokes/min at 25°C for 2 min to obtain a homogeneous suspension. After preparing the appropriate serial dilutions, microbiological parameters were evaluated with the following procedures: total viable count (TVC) and psychrotrophic bacteria count (PTC) were enumerated using PCA at 30°C for 24–48 h and 7°C for 10 days, respectively. Lactic acid bacteria (LAB) were determined by MRS agar incubated at 30°C for 72 h under microaerophilic conditions (anaerobic jar with GasPak system type C). Molds and yeasts (MY) enumeration was performed using RBC agar, and the colonies were counted following a 5‐day incubation at 25°C. All counts were reported as log_10_ CFU/g (Rezaeigolestani et al., 2017).

#### 2.5.2 pH measurement

A homogeneous mixture of each sausage sample and distilled water was prepared and the pH values were measured using a digital pH meter (E520; Metrohm). pH 4 and pH 7 buffers (BDH Laboratory Supplies) were used to calibrate the pH meter (Ansarian et al., 2022).

#### 2.5.3 Evaluation of lipid oxidation

The measurement of lipid oxidation development was conducted using the thiobarbituric acid reactive substances (TBARS) test (Ansarian et al., 2022). Briefly, 10 g of sausage sample was mixed with 1 mL of BHT (0.5% w/v in ethanol) and 35 mL of perchloric acid (4% v/v) and homogenized at 4000 rpm for 2 min. The mixture was filtered through filter paper No. 1, and adjusted to 50 mL by perchloric acid (4%). Five mL of this soluble was mixed with 5 mL of TBA (0.02 M) and heated in 100°C water for 1 h. After cooling, the absorbance was determined using a spectrophotometer at 532 nm. A standard curve was plotted using TEP to calculate malondialdehyde (MDA) values. TBARS values were expressed in mg MDA/kg sausage.

#### 2.5.4 Evaluation of sensory characteristics

A panel of 70 semi‐trained judges, comprising 35 men and 35 women aged 18–40 years, who were non‐smokers, was selected from among students and staff of Zanjan University of Medical Sciences, according to their initial performance in pre‐testing. To ensure a comprehensive evaluation, a preliminary session was held prior to the test during which the panel members could discuss and clarify each attribute of the products to be assessed. The sensory tests were conducted after the panel members agreed on each sensory characteristic of the sausage. Panelists completed their assessments in a private booth under incandescent lighting using unsalted crackers and 25°C water to clean the palate between trials. The samples were randomly coded and a semi‐blind method was used for evaluation. The sensory attributes were reported according to various expressions: light to dark for color; fatness, acid taste, and saltiness for taste; and imperceptible to extremely putrid/off‐odor for odor. Based on the 9‐point hedonic scale method, scores of 1–3.9, 4–6.9, and 7–9 were considered unacceptable, moderately acceptable, and high acceptable limits, respectively (Aminzare et al., 2018). Odor, color, and overall acceptability test sessions were held on days 0, 10, 20, 30, and 40, but taste attribute was evaluated only on days 0, 10, and 20. Also, informed consent was obtained from the panelists to perform these experiments. The institutional ethics committee approved the present project (code of ethics: IR.ZUMS.REC.1398.0146).

### 2.6 Inoculation and enumeration of *L. monocytogenes*

The mortadella sausage slices were sprayed with ethanol 70% (v/v) and each sterile slice was inoculated with 10^4^ CFU/g viable cells of *L. monocytogenes*. Then, the films were placed between two sausage slices and stored under aerobic conditions at 4°C. In order to count the bacteria at the appropriate time intervals, 12.5 g of sausage sample was added to 112.5 mL of 0.1% PW, and serial dilutions (1:10) were prepared after homogenization in a stomacher device. Aliquots of 100 μL of each dilution were cultured on Listeria Chrom agar and were incubated at 37°C for 24 h under aerobic conditions. The enumerations of *L. monocytogenes* were conducted at 0, 10, 20, 30, and 40 days and results were reported as log_10_ CFU/g (Abbasi et al., 2020).

### 2.7 Statistical analysis

A general batch of each experimental group (9 groups for release of phenolic compounds test, and 10 groups for other experiments) was prepared, from which three samples (technical replicates) were randomly selected at each time interval (5 times) for each experiment separately. Results were reported as “Mean ± SE” and statistical analysis of data was performed with SPSS software (Version 18.0 for Windows; SPSS Inc.). All the collected data, including phenolic compound release rate, TVC, LAB, PTC, pH, TBARS, and inoculated *L. monocytogenes* count, were subjected to a two‐way analysis of variance (ANOVA) to test the effects of two fixed factors: treatments (levels: CON, ALG, RES_1_, RES_2_, THY_1_, THY_2_, RES_1_ + THY_1_, RES_2_ + THY_1_, RES_1_ + THY_2_, and RES_2_ + THY_2_) and time (levels: 0, 10, 20, 30, and 40 days). In the sensory analysis, treatments and time were considered as fixed factors, while the panelists' group was considered as a random variable. The model also incorporated the interaction between the fixed and random variables. Tukey test was also used to determine significant differences between data. In all stages of the analysis, a significant level of = 0.05 was considered. In addition, the correlation coefficients between the data were calculated using the Pearson test.

## 3 RESULTS AND DISCUSSION

### 3.1 Release of phenolic compounds from sodium alginate films into mortadella sausage

**FIGURE 1:**
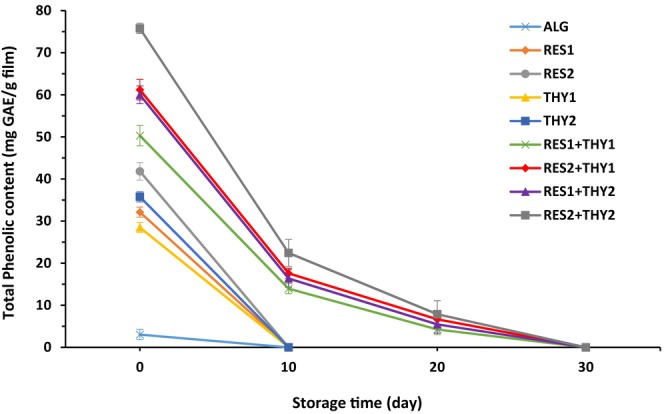
Release of phenolic compounds from sodium alginate films (mg GAE/g film) containing different concentrations of resveratrol and/or thymol into mortadella sausage during 40 days of storage at 4°C. Data are expressed as mean ± SE (*n* = 3).

Direct addition of bioactive compounds to foods causes their rapid consumption and subsequent cessation of their protective effects during processing or storage. Utilizing active packaging systems that incorporate natural preservatives has emerged as a highly effective approach to address this issue because the bioactive compounds are released into the food at a controlled rate. In this regard, the retention time of bioactive agents in the film is an important issue (Charles et al., 2021). Thus, due to the phenolic structure of the bioactive compounds used in this study, it is essential to estimate the release of these compounds from the sodium alginate film matrix to the sausage. As shown in Figure 1, the phenolic contents of all films ranged from 3.03 to 75.76 mg GAE/g film at the beginning of the storage period, and the RES_2_ + THY_2_ treatment had the highest phenolic content. The results of statistical analysis (Table 2) revealed that the addition of RES and/or THY to sodium alginate films significantly increased phenolic contents (*p* ≤ .05). All films showed a decreasing trend in phenolic contents during the storage time (*p* ≤ .05). The films containing RES or THY alone released their phenolic compounds completely within the first 10 days, while the films containing a combination of both compounds took longer time to achieve that level (up to 30 days). In a study conducted by Ruiz‐Navajas et al. (2015) that examined the release of phenolic compounds from chitosan films formulated with two thymus essential oils (containing THY as the main phenolic compound) on cooked ham during 21 days of storage at 4°C, similar findings were observed. They found that during the first 7 days of the storage period, most of the phenolic compounds were released in the meat product. This confirmed a similar release pattern of phenolic compounds from chitosan film containing *Zataria multiflora* Boiss essential oil (containing THY as the main phenolic compound) and grape seed extract to mortadella‐type sausage during refrigerated storage (Moradi et al., 2011).

Bioactive compounds' migration from edible films into meat products depends on several factors, such as electrostatic interactions between polymer chains and bioactive compounds, type and polarity of materials, environmental conditions, structural changes, and ionic osmosis induced by the presence of bioactive compounds, and food composition (Ruiz‐Navajas et al., 2015). In this regard, RES and THY are well released in high‐fat food products (such as cooked sausages) due to their lipophilic properties. In addition, it is supposed that the hydroxyl groups (OH) present in RES and THY can form hydrogen bonds with the carboxylic groups (COOH) existing in sodium alginate. These interactions could potentially modify the stability of the sodium alginate film structure and impact the release of phenolic compounds from the film (Chen et al., 2021).

### 3.2 Effects of sodium alginate films on the shelf life of beef mortadella sausage

#### 3.2.1 Spoilage‐related microbiological changes

##### Total viable count

Meat products are perishable foods that necessitate appropriate intervention to prevent the growth of spoilage‐related microorganisms in order to enhance their shelf life (Umaraw et al., 2020). The microbial changes in the sliced mortadella sausages wrapped with sodium alginate films containing different concentrations of RES and/or THY during 40 days of storage at 4°C are shown in Table 3. The initial TVC range for all experimental groups was 3.65–3.78 log_10_ CFU/g. Cooked sausage is considered sterile after the cooking process and before opening the initial casing. However, the slicing process at the level of retail markets causes microbial contamination of the sausage due to the lack of suitable cold‐chain and hygienic conditions of the environment, equipment, and personnel working in the slicing area (Tajbakhsh et al., 2023). TVC of all sausage samples gradually increased during the 40 days of storage (*p* ≤ .05) until reaching a range of 6.01–8.17 log_10_ CFU/g. The sodium alginate films containing 1% THY (alone or combined with different concentrations of RES) exhibited higher antimicrobial effects against TVC than other treatments at the end of the storage period (*p* ≤ .05). According to the information in Table 4, RES_2_ + THY_2_ treatment had the greatest antibacterial effect with a 2.01 log_10_ cycle reduction in TVC compared to the CON group (*p* ≤ .05). TVC is usually used as an indicator to determine the microbial shelf life of meat products (Korte et al., 2023). Considering the value of 6.7 log_10_ CFU/g as the acceptable TVC limit of emulsion‐type sausage (Korte et al., 2023), only the THY_2_, RES_1_ + THY_2_, and RES_2_ + THY_2_ treatments maintained the microbial shelf life of the product. In agreement with our results, a similar TVC trend was observed by Moradi et al. (2011) and Ruiz‐Navajas et al. (2015), who evaluated the antimicrobial effects of chitosan films containing *Zataria multiflora* Boiss and different thyme essential oils on TVC of mortadella sausage and cooked cured ham during 21 days storage at 4°C, respectively. They stated that the antimicrobial effects were related to thymol as the main component of the essential oils. Liu and Liu (2020) showed similar results regarding the effect of chitosan coating containing THY on TVC of fresh pork during 12 days of storage at 4°C. Karimi‐Khorrami et al. (2022) reported a higher antibacterial effect of alginate‐based films containing THY nanostructures compared to the control group on the TVC of ground meat during refrigeration. Moreover, similar TVC trends were reported in pectin coating incorporated with RES on pork loin (Xiong et al., 2020), alginate coating incorporated with RES on fish fillet (Bazargani‐Gilani, 2018), gelatin–chitosan coating incorporated with RES on fresh beef (Zou et al., 2022), and surface addition of RES along with chitosan and alginate coatings on fish meat (Martínez et al., 2018). The antibacterial properties of THY have been attributed to various mechanisms, including breaking down the outer membrane of microbial cells, increasing 1‐N‐phenylnaphthylamine uptake and lipopolysaccharide release, modifying the composition of fatty acids and phospholipids, affecting genetic material synthesis, permeabilizing and depolarizing the cytoplasmic membrane, and inducing leakage of potassium ions, protons, and ATP channels, resulting in the depletion of membrane potential and hindered energy metabolism (Posgay et al., 2022). The antimicrobial effects of RES are due to several mechanisms such as membrane oxidative damage, DNA damage, the inhibition of enzymes involved in the electron transport chain, and cell division disruption (Sharifi‐Rad et al., 2022). Also, *cis‐* and *trans*‐isomers of RES show different antimicrobial activity. In this study, *trans*‐resveratrol was used, which is effective against both Gram‐positive and Gram‐negative bacteria (Xiong et al., 2020).

**TABLE 2:** Microbial changes (log_10_ CFU/g) in mortadella sausages packaged with sodium alginate films containing different concentrations of resveratrol and/or thymol during 40 days of storage at 4°C.

Microorganisms	Treatments	Storage time (days)				
		0	10	20	30	40
Total viable count	CON	3.78 ± 0.09	6.19 ± 0.05	7.30 ± 0.09	7.62 ± 0.09	8.17 ± 0.01
	ALG	3.77 ± 0.04	6.04 ± 0.05	7.16 ± 0.08	7.44 ± 0.03	7.99 ± 0.10
	RES_1_	3.77 ± 0.07	5.24 ± 0.13	6.35 ± 0.06	6.84 ± 0.04	7.67 ± 0.03
	RES_2_	3.75 ± 0.03	5.15 ± 0.08	6.20 ± 0.05	6.59 ± 0.15	7.34 ± 0.09
	THY_1_	3.72 ± 0.03	4.37 ± 0.07	5.53 ± 0.12	6.08 ± 0.07	6.96 ± 0.11
	THY_2_	3.70 ± 0.11	3.92 ± 0.08	4.45 ± 0.02	5.40 ± 0.01	6.35 ± 0.03
	RES_1_ + THY_1_	3.72 ± 0.06	4.26 ± 0.04	5.34 ± 0.07	5.95 ± 0.08	6.78 ± 0.15
	RES_2_ + THY_1_	3.65 ± 0.09	4.18 ± 0.06	5.20 ± 0.05	5.70 ± 0.26	6.71 ± 0.07
	RES_1_ + THY_2_	3.67 ± 0.03	3.91 ± 0.08	4.36 ± 0.06	5.19 ± 0.06	6.21 ± 0.04
	RES_2_ + THY_2_	3.69 ± 0.05	3.84 ± 0.04	4.32 ± 0.10	5.14 ± 0.08	6.01 ± 0.05
Lactic acid bacteria	CON	ND	4.96 ± 0.06	6.14 ± 0.05	6.41 ± 0.03	7.11 ± 0.03
	ALG	ND	4.87 ± 0.07	6.04 ± 0.09	6.30 ± 0.02	7.01 ± 0.04
	RES_1_	ND	4.17 ± 0.05	5.41 ± 0.03	5.89 ± 0.10	6.83 ± 0.06
	RES_2_	ND	4.06 ± 0.04	5.10 ± 0.02	5.62 ± 0.07	6.63 ± 0.03
	THY_1_	ND	3.94 ± 0.07	4.85 ± 0.06	5.40 ± 0.02	6.43 ± 0.18
	THY_2_	ND	3.75 ± 0.05	4.22 ± 0.02	4.85 ± 0.06	5.83 ± 0.10
	RES_1_ + THY_1_	ND	3.87 ± 0.09	4.80 ± 0.15	5.17 ± 0.06	6.19 ± 0.05
	RES_2_ + THY_1_	ND	3.80 ± 0.02	4.54 ± 0.05	5.01 ± 0.03	6.07 ± 0.05
	RES_1_ + THY_2_	ND	3.65 ± 0.09	4.15 ± 0.07	4.78 ± 0.08	5.42 ± 0.05
	RES_2_ + THY_2_	ND	3.56 ± 0.04	4.11 ± 0.07	4.69 ± 0.04	5.37 ± 0.08
Psychrotrophic bacteria	CON	ND	4.23 ± 0.13	5.23 ± 0.03	6.29 ± 0.09	7.23 ± 0.04
	ALG	ND	4.00 ± 0.08	5.02 ± 0.07	6.08 ± 0.11	7.16 ± 0.06
	RES_1_	ND	3.72 ± 0.13	4.59 ± 0.21	5.72 ± 0.04	6.35 ± 0.09
	RES_2_	ND	3.62 ± 0.07	4.39 ± 0.05	5.57 ± 0.01	6.23 ± 0.04
	THY_1_	ND	3.56 ± 0.04	4.26 ± 0.11	5.16 ± 0.01	5.50 ± 0.05
	THY_2_	ND	ND	4.10 ± 0.04	4.82 ± 0.06	5.18 ± 0.04
	RES_1_ + THY_1_	ND	ND	4.18 ± 0.04	5.09 ± 0.07	5.45 ± 0.01
	RES_2_ + THY_1_	ND	ND	4.13 ± 0.07	4.98 ± 0.04	5.40 ± 0.01
	RES_1_ + THY_2_	ND	ND	4.04 ± 0.08	4.71 ± 0.07	5.14 ± 0.04
	RES_2_ + THY_2_	ND	ND	3.95 ± 0.05	4.63 ± 0.03	5.08 ± 0.02

*Note*: Data are expressed as mean ± SE (*n* = 3).

##### Lactic acid bacteria

LAB are recognized as the main group of spoilage‐related bacteria in refrigerated cooked meat products and can significantly affect their microbial quality. They can grow in the presence of nitrite, smoke, and relatively high concentrations of sodium chloride. Thus, LAB strains may survive during heat treatment or are more likely to be added via post‐processing operations like slicing, packaging, cooling, and refrigeration in cured meat products and emulsion‐type cooked sausages (Rezaeigolestani et al., 2017). Spoilage of meat products with these organisms is often associated with the creation of sour off‐flavor and, in some cases, leads to slime formation, greening, and hydrogen sulfide production (Pothakos et al., 2015). According to Table 3, the LAB populations of all experimental groups were below the detectable level at the beginning of the storage period, which was consistent with previous studies (Hastaoğlu et al., 2021; Rezaeigolestani et al., 2017). LAB populations were observed in all the treatments in day 10 samples (ranging from 3.56 to 4.96 log_10_ CFU/g). LAB levels of all sausage samples increased significantly during 40 days of storage (*p* ≤ .05) until reaching a range of 5.37–7.11 log_10_ CFU/g at the end of the storage period. According to Rezaeigolestani et al.'s (2017) study, LAB count above 7 log_10_ CFU/g in cooked sausages was considered an inappropriate level for human consumption. Only CON and ALG treatment groups exceeded that limit at the end of the storage period. All sodium alginate treatments containing 1% THY, either individually or in combination with RES, showed higher antimicrobial effects than other treatments (*p* ≤ .05). According to the information in Table 4, RES_2_ + THY_2_ treatment had the highest antibacterial effect with a 1.72 log_10_ cycle reduction in the LAB population compared to the CON group (*p* ≤ .05). In accordance with our results, similar LAB trends have been reported in studies conducted on meat products packaging systems enriched by THY or essential oils containing THY as a main antimicrobial compound (Gedikoğlu, 2022; Karimi‐Khorrami et al., 2022; Mohajer et al., 2021). Moreover, sodium alginate treatments containing RES alone had no significant effect on LAB growth (*p* > .05), which is consistent with previous findings about the surface addition of RES along with chitosan and alginate coatings on fish meat (Martínez et al., 2018). However, some studies have reported the potential of *trans*‐RES to damage the cell membrane integrity of LAB (Bazargani‐Gilani, 2018). In this regard, Bazargani‐Gilani (2018) reported lower antibacterial effects of sodium alginate coating containing RES on the LAB population of fish meat compared to other spoilage bacterial groups. They attributed this phenomenon to the ability of LAB to generate ATP and deal with osmotic stress conditions.

Currently, the role of LAB in the spoilage of meat and meat products is still controversial. On one side, several species can release odor‐impact molecules, which may alter the sensory profile of raw or cooked meat. The spoilage character of some LAB taxa is ambiguous and probably correlated with specific spoilage‐associated capacities of individual strains that cannot be attributed collectively to the respective species. Moreover, for the LAB with a negligible role in sensory spoilage, a bioprotective function in meat can be hypothesized as they can provide favorable antagonistic activity against other undesired microorganisms by producing bacteriocins and/or organic acids (Pothakos et al., 2015).

##### Psychrotrophic bacteria

Psychrotrophic bacteria indicate unhygienic conditions during the production and handling of meat products in retail stores. The presence of psychrotrophic bacteria in sausage may be due to the contact of the products with various sources of microbial contamination, including personnel's clothes and hands, the air in storage and processing environments, and water used for washing equipment. The spoilage‐related psychrotrophic bacteria adhere to the surfaces of meat products and compete with other microorganisms (Wei et al., 2019). Changes in PTC of mortadella sausages during 40 days of storage at 4°C are shown in Table 3. The PTC of all experimental groups was below the detectable level at the beginning of the storage period, which was consistent with previous studies (Rezaeigolestani et al., 2017). The PTC values were detectable from the 10th day in CON‐, ALG‐, RES_1_‐, and RES_2_‐treated groups. Furthermore, the PTC values were detectable from the 20th day for other experimental groups. PTC values increased significantly during the rest of storage (*p* ≤ .05) until reaching a range of 5.08–7.23 log_10_ CFU/g at the end of storage. All sodium alginate treatments containing 1% THY, alone or in combination forms, showed higher antimicrobial activities than other treatments against PTC. According to the information in Table 4, RES_2_ + THY_2_ treatment had the highest antibacterial effect with a 1.19 log_10_ reduction compared to the CON (*p* ≤ .05). Similar PTC trends were observed in cooked sausage wrapped with poly lactic acid nanocomposite films containing *Zataria multiflora* Boiss essential oil (Rezaeigolestani et al., 2017), fish meat coated with gelatin containing THY (Mohajer et al., 2021), mortadella sausage containing THY (Hastaoğlu et al., 2021), fish fillet coated with alginate containing RES (Bazargani‐Gilani, 2018), and fish meat coated with chitosan and alginate containing RES (Martínez et al., 2018).

##### Molds and yeasts

Fungi are a global concern for the meat industry due to their ability to grow and multiply in production, distribution, retail, and storage areas. They cause discoloration, off‐odor, and off‐flavor, leading to rejection by consumers. Moreover, surface molds generate mycotoxins and other secondary metabolites that exhibit carcinogenic, degenerative, and toxicogenic effects (Korte et al., 2023; Pateiro et al., 2021). In the present study, no MY were found in any of the experimental groups until the end of storage time. Other similar studies have also reported the absence of MY growth in cooked sausages (Aminzare et al., 2018; Hastaoğlu et al., 2021; Rezaeigolestani et al., 2017; Ruiz‐Navajas et al., 2015). This phenomenon is probably due to the high amounts of sodium chloride in the product, the effectiveness of the heat treatment, and in the case of MY, the non‐recontamination of the product due to the aseptic conditions of the slicing process (Aminzare et al., 2018; Rezaeigolestani et al., 2017; Ruiz‐Navajas et al., 2015).

#### 3.2.2 pH changes

**FIGURE 2:**
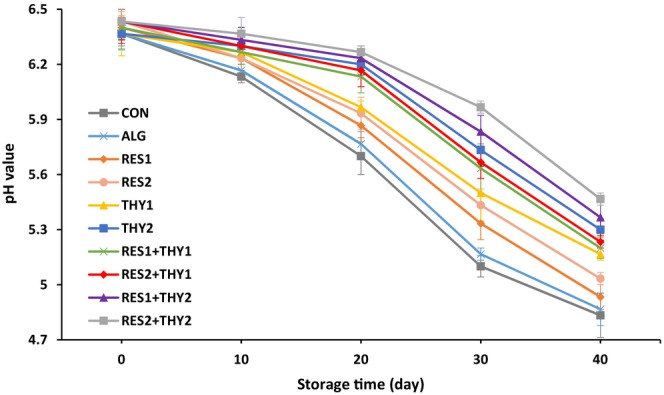
Changes in pH levels of mortadella sausages packaged with sodium alginate films containing different concentrations of resveratrol and/or thymol during 40 days of storage at 4°C. Data are expressed as mean ± SE (*n* = 3).

pH value can be considered as an indicator of hygienic quality and spoilage of meat products (Hastaoğlu et al., 2021). pH changes in mortadella sausages wrapped with sodium alginate films containing different concentrations of RES and/or THY during 40 days of storage at 4°C are shown in Figure 2. The initial pH range of all experimental groups was 6.37–6.43, which was in agreement with the initial pH values of cooked sausages in previous studies (Aminzare et al., 2018; Hastaoğlu et al., 2021). The pH of all sausage samples decreased significantly during storage (*p* ≤ .05) until reaching a range of 4.83–5.47 at the end of storage. CON and RES_2_ + THY_2_ groups had the highest and lowest pH reduction compared to other experimental groups during the storage period, respectively (pH difference of 0.47 according to Table 2). This phenomenon is probably due to the higher growth of LAB and the subsequent production of more lactic acid in the CON sample than in other treatments. All sodium alginate treatments containing 1% THY, whether used individually or in combination with RES, exhibited a lower reduction in pH compared to the other experimental groups (*p* ≤ .05). This observation is likely attributed to the notable bactericidal effects of 1% THY against the LAB population, which is also consistent with the findings presented in Lactic acid bacteria section. Based on the Pearson test, the correlation coefficient (*r*) between LAB populations and pH levels of all samples was .867 on the last day of storage (*p* < .01). This strong linear correlation suggests that LAB growth may be the main cause of pH reduction in mortadella sausages. In this regard, Hastaoğlu et al. (2021) have attributed the lower pH decreasing trend in mortadella sausages containing THY compared to the CON group to the inhibitory effects of THY against LAB growth. Other studies have also concluded a similar concept about cooked sausages wrapped with active packaging films enriched by essential oils containing THY as a main antimicrobial compound (Khodayari et al., 2019; Ruiz‐Navajas et al., 2015).

**TABLE 3:** The mean differences in phenolic compounds released from different sodium alginate films, as well as the pH and TBARS values of mortadella sausages wrapped with various sodium alginate films when compared together during 40 days of storage.

Parameters	Experimental groups	Mean difference								
		ALG	RES_1_	RES_2_	THY_1_	THY_2_	RES_1_ + THY_1_	RES_2_ + THY_1_	RES_1_ + THY_2_	RES_2_ + THY_2_
Release of phenolic compounds (mg GAE/g film)	ALG		29.09*	38.78*	25.45*	32.72*	19.79*	25.45*	24.24*	32.32*
	RES_1_			9.69*	3.63	3.63	9.29*	3.63	4.84	3.23
	RES_2_				13.33*	6.06	18.98*	13.33*	14.54*	6.46
	THY_1_					7.27	5.65	0.00	1.21	6.86
	THY_2_						12.92*	7.27	8.48*	0.40
	RES_1_ + THY_1_							5.65*	4.44	12.52*
	RES_2_ + THY_1_								1.21	6.86*
	RES_1_ + THY_2_									8.08*
pH	CON	0.04	0.12	0.18*	0.22*	0.35*	0.30*	0.33*	0.41*	0.47*
	ALG		0.08	0.14	0.18*	0.31*	0.26*	0.29*	0.37*	0.43*
	RES_1_			0.06	0.10	0.22*	0.17*	0.20*	0.28*	0.34*
	RES_2_				0.04	0.16*	0.11	0.14	0.22*	0.28*
	THY_1_					0.12	0.07	0.10	0.18*	0.24*
	THY_2_						0.05	0.02	0.06	0.12
	RES_1_ + THY_1_							0.03	0.11	0.17*
	RES_2_ + THY_1_								0.08	0.14
	RES_1_ + THY_2_									0.06
TBARS (mg MDA/kg sample)	CON	0.09	0.66*	0.77*	0.49*	0.58*	0.89*	1.12*	1.04*	1.16*
	ALG		0.57*	0.68*	0.40*	0.49*	0.80*	1.03*	0.95*	1.07*
	RES_1_			0.10	0.17*	0.07	0.23*	0.45*	0.37*	0.49*
	RES_2_				0.28*	0.18*	0.12*	0.34*	0.26*	0.38*
	THY_1_					0.09	0.40*	0.62*	0.54*	0.67*
	THY_2_						0.31*	0.53*	0.45*	0.57*
	RES_1_ + THY_1_							0.22*	0.14*	0.26*
	RES_2_ + THY_1_								0.08	0.04
	RES_1_ + THY_2_									0.12*

* Indicate a statistically significant difference (*p* ≤ .05) (*n* = 3).

#### 3.2.3 Lipid oxidation

**FIGURE 3:**
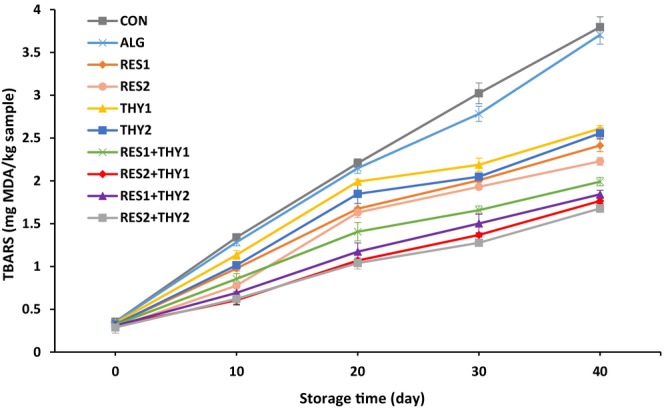
Changes in TBARS levels (mg MDA/kg sample) of mortadella sausages packaged with sodium alginate films containing different concentrations of resveratrol and/or thymol during 40 days storage at 4°C. Data are expressed as mean ± SE (*n* = 3).

Lipid oxidation significantly affects the quality characteristics and shelf life of meat products. The accumulation of secondary products from lipid oxidation can increase product rancidity, which greatly reduces the sensory quality and affects consumer acceptance (Alirezalu et al., 2021). Figure 3 indicates the TBARS changes in mortadella sausages wrapped with sodium alginate films containing different concentrations of RES and/or THY during 40 days of storage at 4°C. At the beginning of the storage, TBARS values of all experimental groups were in the range 0.29–0.35 mg MDA/kg sample, which was in line with previous findings about the initial TBARS value of cooked sausages (Alirezalu et al., 2021). TBARS values of all sausage samples gradually increased during 40 days of storage (*p* ≤ .05) until reaching a range 1.68–3.80 mg MDA/kg sample. According to the information in Table 2, the sodium alginate films containing the combination of 0.004% RES and different concentrations of THY showed higher antioxidant effects than other experimental groups (*p* ≤ .05). Moreover, RES_2_ + THY_2_ treatment had the greatest antioxidant effect with a 1.16 mg MDA/kg sample reduction in TBARS value compared to the CON group. TBARS value is considered a typical indicator of rancidity resulting from lipid oxidation in meat products (Aheto et al., 2020). Some studies suggested 1 mg MDA/kg sample as an acceptable threshold of TBARS value in meat and meat products (Hastaoğlu et al., 2021). However, some other studies stated 2–2.5 mg MDA/kg sample as the maximum acceptable level of TBARS value without any rancidity in the meat products (Alirezalu et al., 2021; Zhang et al., 2019). All samples treated with sodium alginate films containing single or combined forms of RES did not reach the TBARS threshold of 1 and 2.5 mg MDA/kg sample on the 10th and 40th day of storage, respectively. Consistent with these results, similar TBARS trends were found in mortadella sausage containing THY (Hastaoğlu et al., 2021), mortadella sausage wrapped with chitosan films containing *Zataria multiflora* Boiss essential oil (Moradi et al., 2011), cooked cured ham wrapped with chitosan films containing different thyme essential oils (Ruiz‐Navajas et al., 2015), pork loin coated with pectin containing oregano essential oil and RES (Xiong et al., 2020), fish fillet coated with alginate containing RES (Bazargani‐Gilani, 2018), and smoked fish fillet coated with chitosan and alginate containing RES (Martínez et al., 2018) during cold storage.

The antioxidant properties of RES are attributed to its two phenolic groups, which possess resonance activity that stabilizes free radicals formed on phenolic carbons (Xiong et al., 2020). Furthermore, RES can prevent the progression of oxidative processes by binding to metal ions, demonstrating the chelating activity, and subsequently hindering the formation of hydroxyl radicals. The number and position of RES hydroxyl groups play a crucial role in its antioxidant capacity (Tian & Liu, 2020). On the other hand, the phenolic structure of THY is believed to contribute to its antioxidant properties, allowing it to bind with free radicals and exhibit redox activity. This compound can eliminate hydroxyl free radicals and generate phenoxyl radicals, which are important intermediary species. In addition to its direct antioxidant properties, THY has been shown to enhance the activity of several endogenous antioxidant enzymes including catalase, superoxide dismutase, glutathione‐S‐transferase, and glutathione peroxidase, as well as increase the levels of non‐enzymatic antioxidants such as vitamin E, vitamin C, and reduced glutathione. This leads to an improvement in overall antioxidant status (Salehi, Mishra, Shukla, et al., 2018).

#### 3.2.4 Sensory properties

**FIGURE 4:**
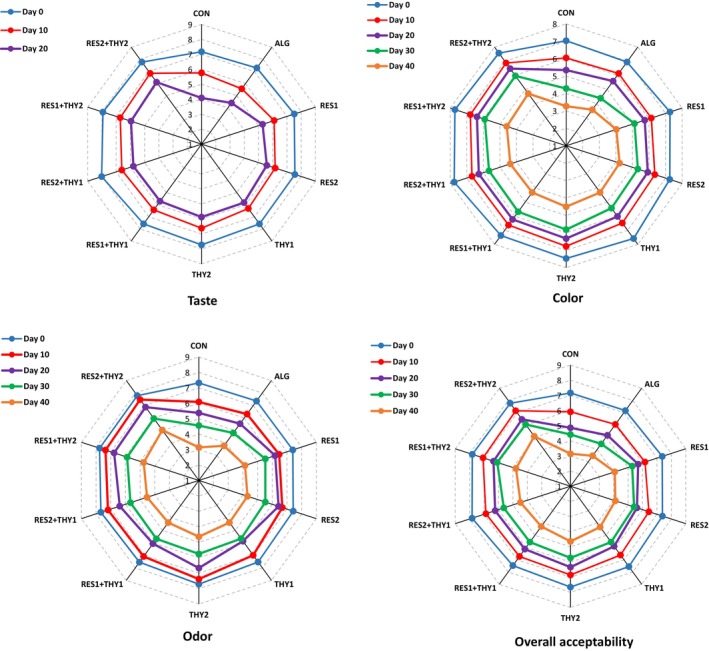
Changes in sensory characteristics of mortadella sausages packaged with sodium alginate films containing different concentrations of resveratrol and/or thymol during 40 days of storage at 4°C (*n* = 3).

Meat products are prone to sensory degradation during the storage period due to their complex matrix, which is mostly composed of proteins, water, and lipids. Therefore, sensory characteristics play a critical role in determining their acceptability (Bolívar‐Monsalve et al., 2019). When producing a new meat product or improving an existing one, even if it is acceptable in terms of microbiological and oxidative quality but does not meet the sensory quality requirements, it cannot be recommended (Hastaoğlu et al., 2021). On the other hand, the intense aroma of bioactive compounds used in food packaging materials may change the sensory properties of sausages (Bolívar‐Monsalve et al., 2019). Figure 4 shows the changes in sensory characteristics of mortadella sausages wrapped with sodium alginate films containing different concentrations of RES and/or THY during 40 days of storage at 4°C. The initial taste, color, odor, and overall acceptability scores of all mortadella sausages were in the range 7.17–8.01, 6.94–7.76, 7.31–7.79, and 7.16–7.83, respectively. All the sensory scores indicated a downward trend during the storage period (*p* ≤ .05). Considering the relatively off‐odor of the samples, the taste attribute was not evaluated from day 20 onwards. Since score 4 was considered an unacceptable threshold in this study, the sensory characteristics of CON and ALG groups were unacceptable, which could be due to spoilage signs such as off‐flavor, discoloration, and slimy texture. Meanwhile, the sensory scores of other treated samples on the same day were in the moderate acceptable range. According to Table 5, the overall acceptability of the CON and ALG groups was significantly lower than that of the other experimental groups (*p* ≤ .05). Additionally, the decreasing trend in the overall acceptability of the RES_1_ + THY_2_‐ and RES_2_ + THY_2_‐treated samples was significantly slower than that of the other experimental groups (*p* ≤ .05). This phenomenon is probably due to the antibacterial and antioxidant properties of RES and/or THY incorporated in sodium alginate films, which delay spoilage signs by suppressing the deteriorative effects of spoilage‐related bacterial growth and lipid oxidation and subsequently prolong the shelf life of the product. According to the Pearson test, the correlation coefficients (*r*) between overall acceptability and the counts of TVC, LAB, and TPC, as well as the TBARS values of all samples, were .540 (*p* < .01), .445 (*p* < .05), .489 (*p* < .01), and .488 (*p* < .01), respectively, on the last day of storage. In confirmation of these results, Hastaoğlu et al. (2021) have reported that THY can prevent microbial spoilage and lipid oxidation in mortadella sausage and thus improve the sensory properties compared to the CON group. In addition, other studies have shown the effects of THY or RES in improving the sensory properties of various meat products during cold storage, including chicken breast fillets immersed in the marinade mixture containing THY (Karam et al., 2019), chicken fillet coated with chitosan solution containing RES and *Satureja bachtiarica* essential oil (containing THY as the main compound) (Abdalbeygi et al., 2022), camel meat wrapped with nanoemulsion‐based basil seed gum film containing RES (Ansarian et al., 2022), fish fillet coated with sodium alginate solution containing RES (Bazargani‐Gilani & Pajohi‐Alamoti, 2020), fresh beef coated with gelatin–chitosan solution incorporated with RES (Zou et al., 2022), and smoked fish fillet coated with chitosan and alginate containing RES (Martínez et al., 2018).

### 3.3 Inhibition of inoculated *Listeria monocytogenes* in mortadella sausage

**FIGURE 5:**
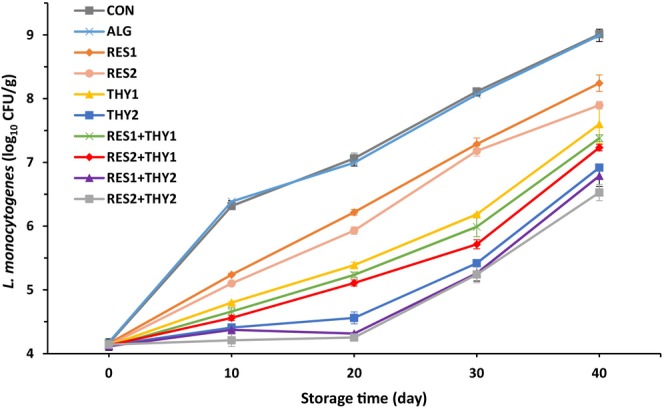
Changes in *L. monocytogenes* population (log_10_ CFU/g) inoculated in mortadella sausages packaged with sodium alginate films containing different concentrations of resveratrol and/or thymol during 40 days of storage at 4°C. Data are expressed as mean ± SE (*n* = 3).

*L. monocytogenes* has the potential to contaminate meat products during processing, distribution, and refrigeration. Contamination of cooked meat products with this bacterium occurs mainly at the post‐processing stages. The ability of *L. monocytogenes* to form biofilms and the challenges associated with removing them from a niche has been identified as contributing factors to its environmental persistence in the food industry (Zamuz et al., 2021). Thus, inadequate sanitary conditions of the equipment used in the slicing process of sausages can lead to contamination by this bacterium (Moradi et al., 2011). Figure 5 shows the changes in the *L. monocytogenes* counts inoculated in mortadella sausages packaged with sodium alginate films containing different concentrations of RES and/or THY during 40 days of storage at 4°C. The initial range of *L. monocytogenes* counts was 4.11–4.18 log_10_ CFU/g. The bacterial counts in all sausage samples increased significantly during storage and reached maximum levels of 6.53–9.01 log_10_ CFU/g on day 40 (*p* ≤ .05). The growth rate of this bacterium was significantly higher in the CON and ALG samples compared to other experimental groups (*p* ≤ .05). All sodium alginate treatments containing THY_2_, in alone or combination forms, showed higher antimicrobial effects than other treatments. According to the information in Table 4, RES_2_ + THY_2_ treatment had the highest antibacterial effect with a 2.05 log_10_ cycle reduction in bacterial population compared to the CON group (*p* ≤ .05). THY induces the dissipation of intracellular metabolites in *L. monocytogenes* by changing the membrane structure and permeability (Liang et al., 2022). RES, in addition to direct antibacterial effects (such as DNA damage, inhibition of enzymes involved in the electron transport chain, and disruption of cell division), probably facilitates the penetration of higher amounts of THY into *L. monocytogenes* cells by disrupting the permeability of the cell membrane (Li et al., 2023). In agreement with these results, Hassan and Cutter (2020) demonstrated the inhibitory effect of a pullulan‐based film enriched with THY against the growth of *L. monocytogenes* inoculated in ready‐to‐eat turkey breast slices during 28 days of storage at 4°C. Pavli et al. (2019) demonstrated a similar antilisterial effect of sodium alginate film containing oregano essential oil on ham slices. Moreover, another study showed a similar inhibitory effect of chitosan edible film containing *Zataria multiflora* Boiss essential oil and grape seed extract against *L. monocytogenes* inoculated in mortadella‐type sausage during 21 days of storage at 4°C (Moradi et al., 2011). In addition, Li et al. (2023) reported the antimicrobial activities of RES against *L. monocytogenes* inoculated in chicken meat.

**TABLE 4:** The average reduction rate in bacterial counts (log_10_ CFU/g) of mortadella sausages wrapped with various sodium alginate films when compared together during 40 days of storage.

Microorganisms	Experimental groups	Mean difference								
		ALG	RES_1_	RES_2_	THY_1_	THY_2_	RES_1_ + THY_1_	RES_2_ + THY_1_	RES_1_ + THY_2_	RES_2_ + THY_2_
Total viable count	CON	0.13	0.63*	0.80*	1.27*	1.84*	1.40*	1.52*	1.94*	2.01*
	ALG		0.50*	0.67*	1.14*	1.71*	1.27*	1.39*	1.81*	1.88*
	RES_1_			0.16	0.63*	1.21*	0.76*	0.88*	1.30*	1.37*
	RES_2_				0.47*	1.04*	0.59*	0.71*	1.14*	1.20*
	THY_1_					0.57*	0.12	0.24*	0.66*	0.73*
	THY_2_						0.44*	0.32*	0.09	0.16
	RES_1_ + THY_1_							0.12	0.54*	0.61*
	RES_2_ + THY_1_								0.42*	0.48*
	RES_1_ + THY_2_									0.06
Lactic acid bacteria	CON	0.09	0.58*	0.79*	0.99*	1.49*	1.14*	1.29*	1.65*	1.72*
	ALG		0.48*	0.70*	0.89*	1.39*	1.04*	1.19*	1.55*	1.62*
	RES_1_			0.21*	0.41*	0.91*	0.56*	0.71*	1.07*	1.14*
	RES_2_				0.19*	0.69*	0.34*	0.49*	0.85*	0.92*
	THY_1_					0.49*	0.14	0.29*	0.65*	0.72*
	THY_2_						0.34*	0.194*	0.16	0.23*
	RES_1_ + THY_1_							0.15	0.50*	0.57*
	RES_2_ + THY_1_								0.35*	0.42*
	RES_1_ + THY_2_									0.06
Psychrotrophic bacteria	CON	0.18*	0.65*	0.79*	1.12*	1.04*	0.84*	0.90*	1.11*	1.19*
	ALG		0.46*	0.60*	0.94*	0.86*	0.65*	0.72*	0.93*	1.01*
	RES_1_			0.14	0.47*	0.39*	0.19*	0.25*	0.46*	0.54*
	RES_2_				0.33*	0.25*	0.04	0.11	0.32*	0.40*
	THY_1_					0.08	0.28*	0.21*	0.01	0.06
	THY_2_						0.20*	0.13	0.06	0.14
	RES_1_ + THY_1_							0.06	0.27*	0.35*
	RES_2_ + THY_1_								0.20*	0.28*
	RES_1_ + THY_2_									0.07
*L. monocytogenes*	CON	0.01	0.70*	0.88*	1.31*	1.84*	1.45*	1.58*	1.96*	2.05*
	ALG		0.69*	0.87*	1.30*	1.83*	1.44*	1.57*	1.95*	2.04*
	RES_1_			0.18*	0.60*	1.14*	0.75*	0.87*	1.25*	1.35*
	RES_2_				0.42*	0.96*	0.57*	0.69*	1.07*	1.17*
	THY_1_					0.53*	0.14	0.27*	0.65*	0.74*
	THY_2_						0.38*	0.26*	0.11	0.21*
	RES_1_ + THY_1_							0.12	0.50*	0.60*
	RES_2_ + THY_1_								0.37*	0.47*
	RES_1_ + THY_2_									0.09

* Indicate a statistically significant difference (*p* ≤ .05) (*n* = 3).

**TABLE 5:** The mean difference in sensory attribute (overall acceptability) of mortadella sausages wrapped with various sodium alginate films when compared together during 40 days of storage.

Sensory attribute	Experimental groups	Mean difference								
		ALG	RES_1_	RES_2_	THY_1_	THY_2_	RES_1_ + THY_1_	RES_2_ + THY_1_	RES_1_ + THY_2_	RES_2_ + THY_2_
Overall acceptability	CON	0.16	0.62*	0.69*	0.89*	1.14*	0.93*	1.10*	1.32*	1.40*
	ALG		0.45*	0.53*	0.72*	0.98*	0.77*	0.94*	1.15*	1.23*
	RES_1_			0.07	0.27*	0.52*	0.31*	0.48*	0.70*	0.78*
	RES_2_				0.19	0.45*	0.24*	0.41*	0.62*	0.70*
	THY_1_					0.25*	0.04	0.21	0.42*	0.50*
	THY_2_						0.21	0.04	0.17	0.25*
	RES_1_ + THY_1_							0.17	0.38*	0.46*
	RES_2_ + THY_1_								0.21	0.29*
	RES_1_ + THY_2_									0.08

* Indicate a statistically significant difference (*p* ≤ .05) (*n* = 3).

## 4 CONCLUSIONS

In the current study, sodium alginate films with different concentrations of RES and/or THY were prepared based on preliminary in vitro trials. Following the release rate assessment of phenolic compounds, their impact on the shelf life and microbial safety of sliced mortadella sausage was examined over a 40‐day storage at 4°C. The sodium alginate films containing 1% THY, either in individual form or combined with RES, exhibited higher antimicrobial effects against spoilage‐related microbial groups and inoculated *L. monocytogenes* when compared to other treatments throughout the storage period. Moreover, the sausage samples wrapped with sodium alginate films containing the combination of 0.004% RES and THY showed lower lipid oxidation trends compared to other experimental groups. The sodium alginate film containing 0.004% RES + 1% THY demonstrated the most pronounced antimicrobial, antioxidant, and sensory effects among all treatments. The present findings provide valuable insights into the potential application of sodium alginate film containing a combination of 0.004% RES and 1% THY as an active packaging material containing natural preservatives in the meat products industry for extending the shelf life and improving the microbial safety of cooked sausages during refrigerated storage. Further research is needed to investigate the limitations of the practical use of this packaging method in order to gather sufficient evidence for their industrial application.

## AUTHOR CONTRIBUTIONS

**Mahsa Hashemi:** Conceptualization (equal); data curation (equal); formal analysis (equal); investigation (equal); methodology (equal); software (equal); validation (equal); visualization (equal); writing – original draft (lead); writing – review and editing (equal). **Majid Aminzare:** Conceptualization (lead); data curation (equal); formal analysis (lead); funding acquisition (lead); investigation (equal); methodology (lead); project administration (lead); resources (equal); software (equal); supervision (lead); validation (equal); visualization (equal); writing – original draft (equal); writing – review and editing (equal). **Hassan Hassanzadazar:** Conceptualization (supporting); formal analysis (equal); funding acquisition (supporting); investigation (equal); methodology (supporting); project administration (supporting); resources (supporting); supervision (supporting); validation (supporting); writing – review and editing (equal). **Shahin Roohinejad:** Conceptualization (supporting); investigation (equal); methodology (supporting); validation (supporting); writing – review and editing (equal). **Reza Tahergorabi:** Conceptualization (supporting); formal analysis (supporting); investigation (equal); methodology (supporting); validation (supporting); writing – review and editing (equal). **Alaa El‐Din Ahmed Bekhit:** Conceptualization (equal); investigation (equal); methodology (supporting); validation (supporting); writing – review and editing (equal).

## CONFLICT OF INTEREST STATEMENT

The authors declare no conflicts of interest for this study.

## Data Availability

The data that support the findings of this study are available on request from the corresponding author. The data are not publicly available due to privacy or ethical restrictions.
